# Social workers’ and their clients’ attitudes concerning control of alcohol use

**DOI:** 10.1186/1940-0640-8-S1-A56

**Published:** 2013-09-04

**Authors:** Elina Renko

**Affiliations:** 1 Department of Social Sciences, University of Helsinki, Helsinki, Finland

## 

This study presents a qualitative analysis of social workers’ and their customers’ attitudes concerning control of alcohol use. Social workers and their customers were asked to comment on the following statements: *Alcohol use is a private affair* and *I believe that social worker can influence customers’ alcohol use*. The analytical focus is on how the two parties commented on the statements and did social workers and their customers do this in a same way or were there differences between them? The current study employs a qualitative attitude research method. The aim of the method is not to make generalizations from sample to population but to analyze plurality of attitude sphere. Social workers (n=14) and their customers (n=14) were interviewed. Interviews were recorded and transcribed. Analysis reveals that both groups commented the statements on three spheres of action. *On an individual sphere*, there were no other actors but an individual customer. The only way to control alcohol use was thus through decisions made by the customer himself. *On a relational sphere*, there were more actors who were seen as having an influence on each other’s. Controlling alcohol use was seen possible by empowering customers or by taking an advantage of networks. *On a structural sphere* the structures of the sphere were seen as guiding the movements of the actors as well as defining their roles and responsibilities. Controlling alcohol use was defined as a task of social worker to remind customers about their responsibilities. Customers presented more comments on the individual sphere, social workers on the structural sphere. The findings suggest that social workers and their customers commented the statements on the similar spheres of action. Further research on comparing social workers’ and customers’ attitudes is warranted.

